# Characterization of Novel Cutaneous Human Papillomavirus Genotypes HPV-150 and HPV-151

**DOI:** 10.1371/journal.pone.0022529

**Published:** 2011-07-25

**Authors:** Anja Kovanda, Boštjan J. Kocjan, Boštjan Luzar, Ignacio G. Bravo, Mario Poljak

**Affiliations:** 1 Institute of Microbiology and Immunology, Faculty of Medicine, University of Ljubljana, Ljubljana, Slovenia; 2 Institute of Pathology, Faculty of Medicine, University of Ljubljana, Ljubljana, Slovenia; 3 Unit of Infections and Cancer, Catalan Institute of Oncology (ICO), L'Hospitalet de Llobregat, Barcelona, Spain; 4 CIBER Epidemiology and Public Health (CIBERESP), Barcelona, Spain; 5 Genomics and Health Centre for Public Health Research (CSISP), Valencia, Spain; Institut Pasteur, France

## Abstract

DNA from two novel HPV genotypes, HPV-150 and HPV-151, isolated from hair follicles of immuno-competent individuals, was fully cloned, sequenced and characterized. The complete genomes of HPV-150 and HPV-151 are 7,436-bp and 7,386-bp in length, respectively. Both contain genes for at least six proteins, namely E6, E7, E1, E2, L2, L1, as well as a non-coding upstream regulatory region located between the L1 and E6 genes: spanning 416-bp in HPV-150 (genomic positions 7,371 to 350) and 322-bp in HPV-151 (genomic positions 7,213 to 148). HPV-150 and HPV-151 are phylogenetically placed within the *Betapapillomavirus* genus and are most closely related to HPV-96 and HPV-22, respectively. As in other members of this genus, the intergenic E2-L2 region is very short and does not encode for an E5 gene. Both genotypes contain typical zinc binding domains in their E6 and E7 proteins, but HPV-151 lacks the regular pRb-binding core sequence within its E7 protein. In order to assess the tissue predilection and clinical significance of the novel genotypes, quantitative type-specific real-time PCR assays were developed. The 95% detection limits of the HPV-150 and HPV-151 assays were 7.3 copies/reaction (range 5.6 to 11.4) and 3.4 copies/reaction (range 2.5 to 6.0), respectively. Testing of a representative collection of HPV-associated mucosal and cutaneous benign and malignant neoplasms and hair follicles (total of 540 samples) revealed that HPV-150 and HPV-151 are relatively rare genotypes with a cutaneous tropism. Both genotypes were found in sporadic cases of common warts and SCC and BCC of the skin as single or multiple infections usually with low viral loads. HPV-150 can establish persistent infection of hair follicles in immuno-competent individuals. A partial L1 sequence of a putative novel HPV genotype, related to HPV-150, was identified in a squamous cell carcinoma of the skin obtained from a 64-year old immuno-compromised male patient.

## Introduction

Papilomaviruses (PVs) are a diverse family of small viruses with a circular double stranded DNA genome, which are etiologically linked with several skin and mucosal epithelial lesions of animals and humans. They are hierarchically classified into categories designated as genera, species and genotypes [1]. To date, full genomes of more than 200 PV genotypes have been publicly deposited, of which roughly 150 have been detected in humans and are referred to as human PVs or HPVs [1], [2]. Currently, HPVs are classified into five genera: *Alpha-*, *Beta-*, *Gamma-*, *Mu-* and *Nupapillomavirus* [1].

According to the 2004 guidelines for PV nomenclature, published by the Study Group of Papillomaviruses of the International Committee on Taxonomy of Viruses (ICTV), in order to be officially recognized as a unique genotype, a candidate PV isolate must differ by at least 10% of its complete gene coding for the major capsid protein (L1 gene) from all other known genotypes, and its complete genome must be sequenced and deposited in the form of clones to the Reference Centre for Papillomaviruses in Heidelberg, Germany [2]. The guidelines issued in 2010 have refrained from strict identity boundaries and have suggested instead the introduction of phylogenetic relationships as a guiding criterion [1]. Traditionally, PV genomic sequences have been obtained directly from epithelial lesions, using cloning methods, which are mainly suitable for characterization of HPV genotypes present in clinical samples in high viral copy numbers [2]. As the field of molecular biology evolved, polymerase chain reaction (PCR), rolling circle amplification, whole genome amplification and, recently, shotgun sequencing have been added to the repertoire of methods used in identification of novel PVs [3], [4]. These technologies have enabled the identification and characterization of many recently identified PVs, especially those present in minute quantities in clinical samples.

Cutaneous HPV genotypes are found within all five PV genera that contain HPVs. They are ubiquitously present in human skin and in the hair follicles of immuno-competent individuals [5]–[7], but can occasionally cause various, predominantly benign skin lesions, including cutaneous warts, e.g. common warts or *verrucae vulgaris* [8]. In hosts with primary immuno-deficiency or with a genetic predisposition, cutaneous HPVs - especially *Betapapillomaviruses* - can cause serious clinical manifestations, such as numerous benign and malignant tumors in patients with the hereditary disease *epidermodysplasia verruciformis* [9]. In immuno-suppressed patients, such as renal transplant recipients, infection with cutaneous HPVs can similarly lead to the development of various benign and malignant skin tumors [10]–[14]. Additionally, several *Betapapillomavirus* HPVs, such as HPV-22, HPV-38, HPV-92 or HPV-96, have been etiologically linked with the development of squamous cell carcinoma of the skin [15]–[18]. Several cutaneous HPVs have also been linked with actinic keratosis, Bowen's disease and non-melanoma skin cancer in connection with UV-damage in immuno-competent hosts [19], [20].

In the last decade, several studies have shown that HPV DNA can be recovered from healthy skin in humans from very early stages of life [6], [21], that skin infections with multiple HPV genotypes are very common [7], [22], [23] and that several recently identified cutaneous HPVs may not be associated with any clinical manifestations [5], [24]. In view of these facts, the clinical significance of any novel HPV genotype needs to be carefully examined.

In this study, two novel HPV genotypes: HPV-150 and HPV-151, isolated originally from eyebrow hair follicles (isolates SIBX1 and SIBX2, respectively) and initially characterized by our group in 2005 [25], were fully cloned, sequenced and characterized. In addition, two quantitative type-specific real-time PCRs were developed and a representative collection of HPV-associated benign and malignant neoplasms and hair follicles was tested in order to assess the tissue predilection and clinical significance of the novel HPV genotypes.

## Materials and Methods

The original isolates of HPV-150 (SIBX1) and HPV-151 (SIBX2) were obtained from eyebrow hair follicles of two immuno-competent males, aged 28 and 25 years, respectively, during our previous study of HPV distribution in eyebrow hair follicles of patients with genital warts [25]. The original SIBX1 positive eyebrow hair follicle sample was used for cloning HPV-150 [25], and a SIBX2 positive sample of scrotal hair, obtained in 2003 from a healthy 23-year-old male individual was used for cloning of HPV-151 [7]. In both cases, DNA was isolated from clinical samples by using a High Pure PCR Template Preparation Kit (Roche Diagnostics GmbH, Mannheim, Germany), according to the manufacturer's instructions.

Whole genome amplification (WGA) of both HPV-150 and HPV-151 was performed prior to PCR amplification, by using an Illustra™ TempliPhi 100 Amplification Kit (GE Healthcare, Amersham, UK), according to the manufacturer's instructions. Following the amplification, the WGA product was diluted 200x fold with PCR-grade H_2_O.

### Amplification, sequencing and cloning of HPV-150

Primers for the reverse long template PCR of HPV-150 were constructed manually on the basis of the previously obtained 373-bp sequence of the HPV-150 L1 gene (GenBank Acc. No AJ606880). A 7,367-bp PCR product of HPV-150 was generated using primer pair X1-longF and X1-longR (Table S1) and the Expand Long Template PCR System (Roche Applied Bioscience, Mannheim, Germany) on a PE9700 Thermo Cycler (Perkin Elmer Applied Biosystems Inc, Foster City, CA).

PCR was carried out in a 25 µl reaction volume containing 5 µl of diluted WGA product, 2.5 µl of 10× Expand Long Template buffer 2 with 27.5 mM MgCl_2_, 500 µM of dNTPs, 0.75 U of Expand Long Template Enzyme Mix, and 3 pmol of each primer. The thermal cycler program was set to 2 min at 94°C, followed by 40 cycles consisting of 10s at 94°C, 30 s at 53°C, and 8 min at 68°C. The final extension step was performed at 68°C for 10 min and the reaction mixtures were then cooled to 4°C.

Initial sequencing of the 7,367-bp HPV-150 PCR fragment was done by primer walking (primers are listed in Table S1) using the ABI Prism® 310 Genetic Analyzer System (Applied Biosystems), and Big Dye® Terminator 1.1 Cycle Sequencing Kit (Applied Biosystems), according to the manufacturer's instructions.

In order to clone the complete genome of HPV-150, two overlapping blunt-end PCR products of sizes 3,465-bp and 4,375-bp were generated using primer pairs X1F18 and X1R18; and X1F5 and X1R5, respectively (Table S1). Both blunt-end PCR products were generated using KOD Xtreme™ Hot Start DNA Polymerase (Toyobo Novagen, EMD Biosciences Inc., San Diego, CA).

PCR was carried out in a 25 µl reaction volume containing 1 µl of diluted WGA product, 12.5 µl of 2× Xtreme Buffer, 500 µM (each) of dNTPs, 0.02 U of KOD Xtreme™ Hot Start DNA Polymerase, and 0.6 pmol of each primer. The thermal cycler program was set to 2 min at 94°C, followed by 40 cycles consisting of 10 s at 94°C, 30 s at 60°C, and 3 to 6 min at 68°C. Following amplification, the reaction mixtures were cooled to 4°C.

Before cloning, PCR products were excised and purified from 1% agarose gel using a GeneJET™ Gel Extraction Kit (Fermentas, Vilnius, Lithuania). Plasmids containing overlapping PCR products of HPV-150 were prepared using a CloneJET™ PCR Cloning Kit (Fermentas), according to the manufacturer's instructions. A TransformAid™ Bacterial Transformation Kit (Fermentas) was used to transform *E.coli* strain JM107 with the HPV-150 clones, according to the manufacturer's instructions.

Plasmid clones isolated from the transformed overnight bacterial culture with a GeneJET™ Plasmid Miniprep Kit (Fermentas) were checked for correct HPV inserts by size analysis and sequencing with pJET1.2 sequencing primers, according to the manufacturer's instructions.

Sequencing by primer walking was carried out for both clones, confirming the full genome sequence of HPV-150. The obtained sequences were additionally confirmed by commercial sequencing of the selected clones at Macrogen Inc. (Seoul, Korea) and at Microsynth AG (Balgach, Switzerland).

All primers used in the study were designed manually and checked for Tm and secondary structures using NetPrimer (Premier Biosoft International) and TIB-Molbiol (Berlin, Germany) free web-based software. The ‘Primer Pair Specificity Checking Parameters’ function of Primer-BLAST (NCBI) was used in order to check for the specificity of the primers.

### Amplification, sequencing and cloning of HPV-151

In the case of HPV-151, the primers initially constructed on the basis of the previously obtained 367-bp sequence of the HPV-151 L1 gene (GenBank Acc. No AJ606879) failed to amplify the remainder of the genome. In order to solve this problem, additional degenerate primers were designed, based on the genome of HPV-22, the genotype which was most closely related to HPV-151. An initial assessment of the primers was done by short PCR using the DreamTaq™ Green PCR Master Mix (2X) (Fermentas UAB - Thermo Fisher Scientific, Vilnius, Lithuania), following the manufacturer's instructions. The amplified DNA was examined by gel electrophoresis and several degenerate primer pairs produced PCR products of the expected size, ranging from 100 to 1,328-bp. Following sequencing of these short amplicons, several additional primers, specific for the novel genotype HPV-151, were designed (Table S1). Finally, a 6,256-bp fragment of the HPV-151 genome was amplified using the primer pair X2-L1For and X2-longR2, designed on the basis of the sequence obtained using degenerate primer pair HPV22-L1f1mix and HPV22-L1-r1mix (Table S1), and the Expand Long Template PCR System on a PE9700 Thermo Cycler.

PCR was carried out in a 25 µl reaction volume containing 1 µl of diluted WGA product, 2.5 µl of 10× Expand Long Template buffer 2 with 27.5 mM MgCl_2_, 500 µM of dNTPs, 0.75 U of Expand Long Template Enzyme Mix and 3 pmol of each primer. The thermal cycler program was set to 2 min at 94°C, followed by 40 cycles consisting of 10 s at 94°C, 30 s at 55°C, and 7 min at 68°C. The final extension step was performed at 68°C for 7 min and the reaction mixtures were then cooled to 4°C.

Initial sequencing of the 6,256-bp HPV-151 PCR fragment was done by primer walking with the primers listed in Table S1.

For cloning HPV-151, two overlapping blunt-end PCR products of sizes 2,785-bp and 5,481-bp were generated using primer pairs X2F17 and X2R17; and X2F3 and X2R3, respectively (Table S1) by using KOD Xtreme™ Hot Start DNA Polymerase (Toyobo Novagen, EMD Biosciences Inc.).

PCR conditions and the entire further cloning and sequencing procedure for HPV-151 were the same as described above for HPV-150.

### ORF and phylogenetic analysis

All partial sequences obtained for HPV-150 and HPV-151 were assembled using ContigExpress of Vector NTI Advance™ v11.0 (Invitrogen, Carlsbad, CA). The open reading frames (ORFs) of HPV-150 and HPV-151 were determined using the ORF finder function of Vector NTI Advance™ v11.0 and were additionally confirmed by genome organization analysis and comparison with closely related HPV genotypes using the BioEdit Sequence Alignment Editor [26].

The phylogenetic relationships of HPV-150 and HPV-151 within the PV family were determined based on the L1 gene. Briefly, L1 nucleic acid sequences were converted to protein sequences and aligned using MAFFT v6.832b (http://mafft.cbrc.jp/alignment/software) [27], reverse translated using Pal2Nal (http://www.bork.embl.de/pal2nal/) [28] and filtered using Gblocks (http://molevol.cmima.csic.es/castresana/Gblocks.html) [29], [30]. Maximum likelihood phylogenetic inference was conducted using RAxML version v7.2.8 [31]–[33] alpha (PTHREADS version) on an 2x CPU 6 core AMD Opteron 2431 with 12 GB RAM DDR2-800 (kindly provided free of charge by ABAKUS plus, Kranj, Slovenia). A generalized time-reversible (GTR) model with four gamma discrete rate categories and three partitions, one per codon position, was used for the analysis. One thousand bootstrap replicates were generated to determine node support for the best tree.

### HPV-150 and HPV-151 type specific real-time PCRs

Primers and a probe specific for HPV-150 were chosen within the L1 gene by using the on-line ProbeFinder software (Roche Applied Science), resulting in a PCR product of 60-bp in length. Primers RT-150-F (5′- AGGCCTTACCTTGTGCTGAA -3′, HPV-150 nucleotide positions 6,355 to 6,374) and RT-150-F (5′- TTAATTCTAAAGGAGGACATGAACC -3′, HPV-150 nucleotide positions 6,390 to 6,414), together with FAM-labeled Universal Probe #50 (Roche Applied Science) were used for HPV-150 specific real-time PCR.

Primers and a probe specific for HPV-151 were chosen within the L1 gene by using the on-line ProbeFinder software (Roche Applied Science), resulting in a PCR product of 60-bp in length. FAM-labeled Universal Probe #127 (Roche Applied Science) was used together with primers RT-151-F (5′- ATCAGCAACAGATGGAGCAA -3′, HPV-151 nucleotide positions 5,838 to 5,857) and RT-151-R (5′- GCTCTATACTGGTTCCCTGACAC -3′, HPV-151 nucleotide positions 5,875 to 5,897) for HPV-151 specific real-time PCR.

The plasmid reference clones of HPV-150 and HPV-151, containing the L1 sequence, were used to optimize the real-time PCR conditions. Plasmid DNA concentration was quantified using a NanoDrop ND-1000 spectrophotometer (Nanodrop Technologies, Oxfordshire, UK) and the number of plasmid copies/µl was calculated using the equation of Whelan et al. [34].

The optimized real-time PCR reaction mixture for both type-specific real-time PCRs contained 10 µl of LightCycler® Probes Master 2x conc. (Roche Applied Science), 0.2 µM of each primer, 0.05 µM of Universal Probe #50 or #127 (Roche Applied Science), 1U of LightCycler® Uracil-N-Glycosylase (Roche Applied Science), sample DNA and water up to a final volume of 20 µl. The assays were performed on a LightCycler® 480 (Roche) under the following conditions: Uracil-N-Glycosylase activation at 40°C and pre-incubation at 95°C for 10 min each, followed by 45 cycles of 95°C for 10 s (denaturation) and 59°C for 30 s (annealing) and 72°C for 1 s (extension). Acquisition of the fluorescence signal was performed in single mode during the annealing step of each cycle. The final cooling step consisted of a 10 s hold at 40°C. The conditions of the real-time PCR for HPV-150 and HPV-151 were intentionally identical, since this allowed simultaneous testing of samples on the LightCycler® 480 (Roche).

To evaluate the linearity of the assays, triplicate ten-fold serial dilutions of the reference plasmids of HPV-150 and HPV-151 were prepared in 1.5 ml DNA LoBind tubes (Eppendorf, Hamburg, Germany) using a water solution with carrier RNA (1 µg/ml). The limits of detection of the assays were determined by testing 15 replicates of the reference plasmid dilutions corresponding to an input of 25, 13, 6, 3, 1.5, 0.8 and 0.4 copies per reaction.

The specificity and cross-reactivity of the assays were tested using 10^7^ to 10^8^ copies of synthetic partial L1 sequences (inserted into plasmid pUC57, by Genscript, Piscataway, USA) of the following *Betapapillomavirus*es: HPV-9, HPV-15, HPV-17, HPV-22, HPV-23, HPV-37, HPV-38, HPV-80, HPV-100, HPV-111, HPV-113, HPV-120, HPV-122, HPV-92 and HPV-96.

### Clinical samples

The tissue predilection and clinical significance of HPV-150 and HPV-151 were assessed on a representative collection of HPV-associated benign and malignant neoplasms and hair follicles. The DNA was isolated from a total of 540 samples obtained from the same number of patients, including tissue samples of histologically confirmed genital warts (71), laryngeal papillomas (78), cervical squamous cell carcinomas (100), common warts (101), squamous cell carcinoma (SCC) of the skin (52), basal cell carcinoma (BCC) of the skin (49) and hair follicles (89), as described previously [7], [25], [35]-[38]. The quality of isolated DNA and absence of PCR inhibitors were checked by amplification of a 110-bp fragment of human beta-globin, as described previously [36]. The number of human cells per µl of clinical sample in samples positive for HPV-150 or HPV-151 was calculated from the beta-globin concentration, as described previously [17], and used to determine the ratio between the number of viral genomes and human cells. All positive results of the HPV-150 and HPV-151 real-time PCRs were confirmed by sequencing.

The presence of additional HPV genotypes in HPV-150 and/or HPV-151 positive samples was determined using two commercial line-probe assays: INNO-LiPA HPV Genotyping Extra (Innogenetics, Gent, Belgium), which allows identification of 28 different *Alphapapillomavirus* genotypes; RHA skin (beta) HPV (Diassay B.V., Rijswijk, The Netherlands), which allows identification of 26 different *Betapapillomavirus* genotypes, and additionally by consensus GP5+/6+ in-house PCR and sequencing of PCR products [39].

### Ethics Statement

None of the 540 clinical samples tested were collected solely for the purpose of this study. All samples included in the study were collected in compliance with the Helsinki Declaration. Samples of cervical cancer, hair follicles, genital warts and common warts were collected prospectively in our previous studies. These studies were approved by the Ethics Committee of the Ministry of Health of the Republic of Slovenia (consent references 34/11/06, 83/11/09, 174/05/09, 97/11/09 and 100/12/09) and written informed consent was obtained from each patient. Samples of laryngeal papillomas, squamous cell carcinoma and basal cell carcinoma of the skin were retrieved from the tissue collection of paraffin-embedded samples of the Institute of Pathology, Faculty of Medicine, University of Ljubljana. Approval from the Institutional Review Board of the Institute of Pathology, Faculty of Medicine, University of Ljubljana was obtained prior to starting work on samples included in this study. In accordance with the national legislation of the Republic of Slovenia, no informed consent is needed for research on archival clinical samples. In order to protect the identity of the patient, all archival clinical samples used in the study were coded and tested anonymously. The only available data were patient gender, age and immune status (if collected during the original study).

## Results and Discussion

Partial L1 sequences of HPV-150 (SIBX1; 373-bp) and HPV-151 (SIBX2, 367-bp) were deposited in GenBank in March 2005 under Acc. Nos. AJ606880 and AJ606879, respectively. The four reference clones, covering the full genomes of HPV-150 and HPV-151, were deposited in March 2010 in the Reference Centre for Papillomaviruses, Heidelberg, Germany, where their sequences were independently confirmed and the two genotypes were officially named in April 2010.

The genome of HPV-150 has a total length of 7,436-bp, with a GC ratio of 39.70%, and is deposited in GenBank under Acc. No. FN677755. The genome of HPV-151 has a total length of 7,386-bp, with a GC ratio of 39.79%, and is deposited in GenBank under Acc. No. FN677756.

**Figure 1:**
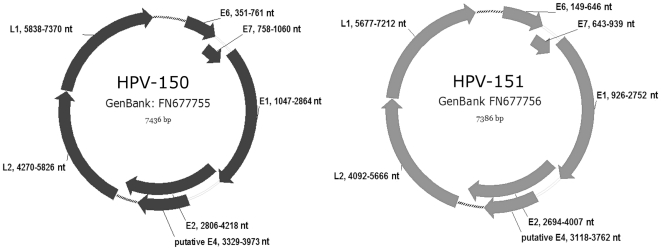
Genomic organization of HPV-150 and HPV-151 showing genomic positions of viral genes E6, E7, E1, E2, L1, L2, and the non-coding regions located between L1 and E6 (LCR); and E2 and L2 (dotted line).

**Figure 2:**
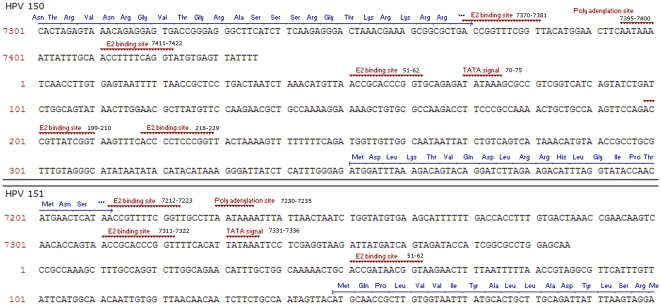
Characteristic features of the long control region (LCR) of HPV-150 and HPV-151, showing genomic locations of E2 binding sites (ACC-N_5-7_-GGT), polyadenilation sites (AATAAA), and TATA signals (TATAAA or TATA(A/T)A(A/T).

ORF analysis and sequence alignment comparisons showed both HPV-150 and HPV-151 to have the typical genomic organization of HPVs and to contain at least six viral genes: E6, E7, E1, E2, L2 and L1, as well as possibly a gene for the E4 protein (Figure 1). In addition to the mentioned ORFs, the genomes of both new genotypes contain a non-coding upstream regulatory region (URR) or long control region (LCR) located between the L1 and E6 genes, spanning 416-bp in HPV-150 (genomic positions 7,371 to 350) and 322-bp in HPV-151 (genomic positions 7,213 to 148) (Figure 2). The LCRs of both HPV-150 and HPV-151 lack the conserved M29 and M33 motifs [40] which are typical for members of *Betapapillomavirus* species 1 [41] but are absent in other *Betapapillomavirus* species [42]. As in other members of *Betapapillomaviruses*, the intergenic region located between E2 and L2 genes is very short (51-bp in HPV-150 (genomic positions 4,219 to 4,269) and 84-bp in HPV-151 (genomic positions 4,008 to 4,091)) and does not encode for an E5 gene.

**Figure 3:**
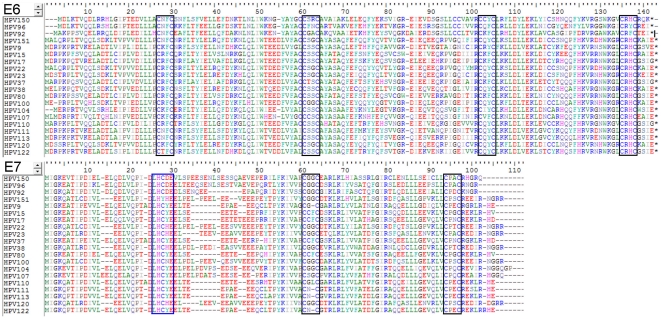
Amino acid alignment of HPV-150 and HPV-151 E6 and E7 proteins with corresponding proteins of closely related genotypes from *Betapapillomavirus* species 2, 4 and 5. The locations of zinc-binding domains (CxxC(x)_29_CxxC) are indicated by black boxes, and the location of the pRb-binding core sequence (LxCxE) is indicated with a blue box.

HPV-150 and HPV-151 contain two regular zinc binding domains (CxxC(x)_29_CxxC) within their E6 protein, at amino-acid positions 24 and 98; and 27 and 101, respectively (Figure 3). Both genotypes also contain one regular zinc binding domain (CxxC(x)_29_CxxC) within their E7 protein, at amino-acid positions 50 and 53, respectively. However only HPV-150 also contains the regular pRb-binding core sequence (LxCxE) at amino-acid position 24 of E7 (Figure 3). Unlike other members of *Betapapillomavirus* species 2, HPV-151 does not contain the regular pRb-binding core sequence (LxCxE) within its E7 protein (Figure 3).

**Figure 4:**
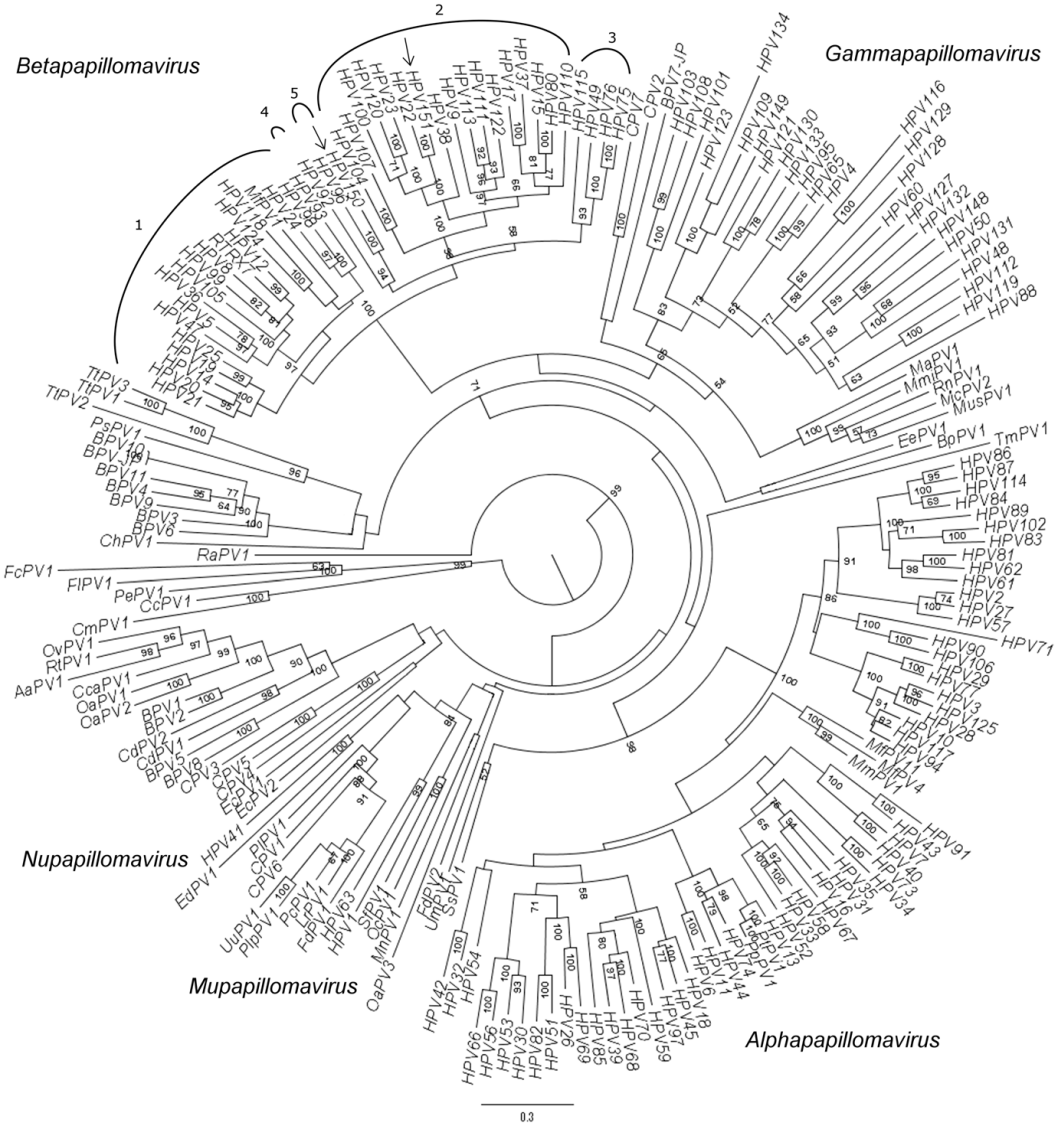
A consensus phylogenetic tree of 200 PV based on nucleotide sequences of the L1 gene. Numbers at nodes show maximum likelihood bootstrap support (%) above 50. *Betapapillomavirus* species numbers are indicated above the corresponding genotypes.

Phylogenetic analyses using the L1 gene sequences of a representative selection of 200 PVs place both HPV-150 and HPV-151 within the *Betapapillomavirus* genus (Figure 4). As shown in Figure 4, the closest relative of HPV-150 is HPV-96 (79.59% nucleotide (nt) identity in the L1 gene; distance in substitutions per site for L1; 0.264440 and 0.103792 for nt and for amino-acid (aa), respectively). HPV-96 belongs to *Betapapillomavirus* species 5 and was originally isolated from a SCC of the skin [18]. The next phylogenetically closest genotype to HPV-150 and HPV-96 is HPV-92, which belongs to *Betapapillomavirus* species 4, and was originally isolated from a BCC of the skin [16]. Since these three genotypes seem to have evolved through intra-host duplication [43] and two of them show at least some carcinogenic potential, the ancestral genotype from which these genotypes were derived may have itself been an oncogenic HPV genotype.

As shown in Figure 4, HPV-151 appears to be phylogenetically nested within *Betapapillomavirus* species 2, with HPV-22 being its closest relative (86.98% nt identity for the L1 gene; 0.164208 and 0.084639 distance in substitutions per site for L1 nt and aa, respectively). HPV-22 was originally isolated from chest macules [15]. Currently, *Betapapillomavirus* species 2 encompasses 17 HPV genotypes isolated from humans, making it one of the largest species among PVs. The generation of such viral diversity has probably occurred through intra-host duplication events, which may reflect multiple ways of succeeding in adaptation to the human host [43]. An example of this capacity to establish infection in multiple ways can be seen from the fact that particular genotypes of *Betapapillomavirus* species 2 have been found in lesions ranging from macules and other benign skin lesions, to actinic keratosis, BCC and SCC of the skin, and even head and neck and esophageal carcinoma, as in the case of HPV-100 [44].

In order to assess the biological and clinical importance of the novel HPV genotypes, two quantitative HPV-150 and HPV-151 type-specific PCR assays were developed. The standard curves of both assays showed an excellent correlation coefficient (R^2^ = 1.00) between the Ct (threshold) and the log of starting viral copy number across at least seven orders of magnitude. The 95% limits of detection determined by Probit analysis using the PASW Statistics 18 program (SPSS GmbH Software, Munich, Germany) were 7.3 copies/reaction (range 5.6 to 11.4) and 3.4 copies/reaction (range 2.5 to 6.0), for the HPV-150 and HPV-151 assays, respectively. For HPV-150 assay, the intra- and inter-assay coefficient of variation (CV (%)  =  (st.dev/mean Ct)×100) were 0.55 and 1.43 for 100 copies per reaction, respectively, and 1.73 and 1.81 for 10 copies per reaction, respectively, and corresponding values for HPV-151 assay were 0.81 and 1.54 for 100 copies per reaction, respectively, and 1.02 and 1.98 for 10 copies per reaction, respectively. Both assays showed no cross-reactivity with any of the tested non-targeted HPV genotypes.

Using the developed type-specific PCRs, a representative collection of HPV-associated benign and malignant neoplasms and hair follicles was tested for the presence of HPV-150 and HPV-151. The sample collection was chosen to represent various sites of HPV infection (mucosal/cutaneous) and possible outcomes of HPV infection (benign/malignant lesions)(Table 1). The panel of samples thus included the most important HPV-associated malignant neoplasms of mucosal (cervical squamous cell carcinoma) and cutaneous (SCC and BCC of skin) origin, and the most important HPV-associated benign neoplasms of mucosal (genital warts, laryngeal papillomas) and cutaneous (common warts) origin. In addition, we tested hair follicles, which are commonly used as the best approximate to skin biopsies, to examine the prevalence of *Betapapillomaviruses* in healthy skin [45], [46] and are known to be a main reservoir of commensal HPV genotypes in immuno-competent individuals [5]–[7].

**Table 1:** Clinical samples tested for the presence of HPV-150 and HPV-151.

Tissue type	Sample type	No. of samples tested	No. (%) of HPV-150 positive samples	No. (%) of HPV-151 positive samples
Mucosal				
	Genital warts	71	0	0
	Laryngeal papillomas	78	0	0
	Cervical cancer	100	0	0
Cutaneous				
	Common warts	101	4 (4.0)	4 (4.0)
	Squamous cell carcinoma of the skin	52	3 (5.8)	1 (1.9)
	Basal cell carcinoma of the skin	49	1 (2.0)	3 (6.1)
Hair follicles				
		89	3 (3.4)	2 (2.2)
Total		540	11 (2.0)	10 (1.9)

As shown in Table 1, none of the 249 mucosal tissue samples tested were positive for HPV-150 and HPV-151, indicating little, if any, mucosal tropism of both novel HPV genotypes.

Of the 101 samples of common warts tested, four samples (4.0%) contained HPV-150, indicating the cutaneous tropism of this genotype (Table 1). Of HPV-150 positive common warts, three samples contained at least one additional HPV genotype (samples 1, 2 and 4, Table 2): HPV-27 from *Alphapapillomavirus* species 4, a well known genotype capable of causing common warts; HPV-5 and HPV-14, cutaneotropic members of *Betapapillomavirus* species 1; and HPV-15 belonging to *Betapapillomavirus* species 2 – all three later being genotypes associated with the development of cutaneous tumors in immuno-suppressed patients or patients with *epidermodysplasia verruciformis*. Due to the presence of more than one HPV genotype in these lesions, the development of common warts could not be conclusively attributed to HPV-150. In contrast, in the fourth HPV-150 positive common wart (sample 3, Table 2), HPV-150 was the only HPV genotype detected, despite the use of several HPV genotyping assays, targeting a range of *Alphapapillomavirus* and *Betapapillomavirus* genotypes, indicating a possible causative role of HPV-150 in the development of this particular common wart. Although the HPV-150 viral load was relatively low (1/31 viral genomes/No. human cells) our finding is in agreement with recent studies which showed that viral load of *Betapapillomaviruses* associated with cutaneous warts is substantially lower than that of *Alphapapillomaviruses* (HPV-3, HPV-27, HPV-57) associated with similar lesions (1/2 to 1/10 000 viral genomes/No. human cells) [47]. However, further research is needed in order to reach any final conclusions on the ability of HPV-150 to cause common warts in absence of other factors.

**Table 2:** Characteristics of HPV-150 positive samples and patients.

Sample type	Sample ID	Histology result	Age	Gender	Viral genomes/No. of human cells	Presence of other HPV genotypes
cutaneous	1	Common wart	15	Male	1/15	HPV-27
cutaneous	2	Common wart	81	Female	1/212	HPV-5, HPV-14, HPV-15
cutaneous	3	Common wart	90	Male	1/31	None
cutaneous	4	Common wart	16	Male	1/12	HPV-5
cutaneous	5	Squamous cell carcinoma of the skin	82	Male	1/57950	None
cutaneous	6	Squamous cell carcinoma of the skin	80	Male	1/9508	HPV-151, HPV-37
cutaneous	7	Squamous cell carcinoma of the skin	86	Male	1/140	HPV-8, HPV-75
cutaneous	8	Basal cell carcinoma of the skin	81	Female	1/22638	HPV-151
hair follicle	9	/	29	Male	Nd	HPV-6, HPV-8, HPV-9, HPV-17, HPV-21
hair follicle	10	/	27	Male	Nd	None
hair follicle	11	/	52	Female	Nd	HPV-8, HPV-14, HPV-38, HPV-93

Nd – Not determined.

As shown in Table 1, HPV-150 was detected in four (4.0%) of the 101 samples of malignant skin tumors tested, further supporting cutaneous tropism of HPV-150. Similarly to the common warts, several additional cutaneous genotypes from *Betapapillomavirus* genus were present in three out of four HPV-150 positive samples of malignant skin tumors included in the study (samples 6–8, Table 2), greatly complicating the interpretation of our findings. The interpretation of our results is additionally complicated by the difference in viral loads observed between an abortive HPV infection leading to malignant transformation of the skin, which typically involves *Betapapillomavirus* genotypes present in minute quantities [13], [18], [48]–[50] and on the other side the productive HPV infection of the skin resulting in benign tumors, such as common warts, which usually harbor viruses in higher concentration [47]. Such differences make it near impossible to distinguish causative or ‘driver’ HPV genotypes from commensal or ‘passenger’ HPV genotypes, also present on the lesion and/or the surrounding skin [48]. Thus, although the HPV-150 was the only HPV genotype detected in a single case of SCC of the skin (sample 5, Table 2), due to its presence in very low quantity (1/57,950 viral genomes/No. human cells), further studies are needed in order to establish if HPV-150 is a causative or commensal genotype in sporadic cases of the SCC of the skin.

As shown in Table 1, HPV-150 was also found in three (3.4%) out of 89 hair follicle samples tested (Table 1), finally confirming cutaneous tropism of HPV-150.

Long term persistent HPV infection is a key requirement for the development of HPV-related anogenital cancers, but its role is less clear in the case of HPV-related skin cancer [13], [20], [48]-[50]. In order to determine whether HPV-150 can establish persistent infection, seven hair follicle samples collected over the course of one year from one of the HPV-150 positive individuals were tested using HPV-150 type-specific PCR. In this immuno-competent 22-year-old male, HPV-150 was detected in all seven hair follicles tested, confirming its potential for persistent infection. However, similarly to the majority of other *Betapapillomavirus* infections in immuno-competent individuals [22]-[24], no obvious epithelial lesions were recorded as a result of the persistence of HPV-150 in our patient during 12 months of follow-up.

Of the 101 samples of common warts tested, four samples (4.0%) contained HPV-151, indicating its cutaneous tropism (Table 1). Of HPV-151 positive common warts, two samples contained at least one additional *Betapapillomavirus* genotype (samples 12 and 14, Table 3): HPV-9, the type species of *Betapapillomavirus* species 2; HPV-19, HPV-36 and HPV-93 of *Betapapillomavirus* species 1; and HPV-49 of *Betapapillomavirus* species 3. Since all listed genotypes have cutaneous tropism and are etiologically associated with the development of cutaneous tumors in immuno-suppressed patients or patients with *epidermodysplasia verruciformis*, these two common warts could not conclusively be attributed etiologically to HPV-151. In contrast, two HPV-151 positive common wart samples (samples 13 and 15, Table 3) did not contain any additional HPV genotypes, indicating a possible causative role of HPV-151 in the development of these two common warts. Both samples had viral loads (1/493 and 1/103 viral genomes/No. human cells, respectively) of magnitude characteristic for *Betapapillomavirus* genotypes in common warts [47], however the final contribution of HPV-151 to the development of these lesions requires further studies.

**Table 3:** Characteristics of HPV-151 positive samples and patients.

Sample type	Sample ID	Histology result	Age	Gender	Viral genomes/No. of human cells	Presence of other HPV genotypes
cutaneous	12	Common wart	67	Male	1/19	HPV-9, HPV-93
cutaneous	13	Common wart	76	Male	1/439	None
cutaneous	14	Common wart	55	Female	1/168	HPV-19, HPV-36, HPV-49
cutaneous	15	Common wart	74	Female	1/103	None
cutaneous	6	Squamous cell carcinoma of the skin	80	Male	1/1994	HPV-150, HPV-37
cutaneous	16	Basal cell carcinoma of the skin	84	Female	1/691	None
cutaneous	17	Basal cell carcinoma of the skin	84	Female	1/39	HPV-21, HPV-36, HPV-24
cutaneous	8	Basal cell carcinoma of the skin	81	Female	1/113	HPV-150
hair follicle	18	/	26	Male	Nd	HPV-5, HPV-19, HPV-21, HPV-22, HPV-24, HPV-38, HPV-93
hair follicle	19	/	33	Male	Nd	HPV-23, HPV-93

Nd – Not determined.

As shown in Tables 1 and 3, HPV-151 was detected in four (4.0%) of the 101 malignant skin tumor samples and in three (3.4%) of 89 hair follicle samples tested, finally confirming the cutaneous tropism of this novel HPV genotype. In the majority of the HPV-151 positive samples, several other HPV genotypes were identified (Table 3).

In the single HPV-151 positive BCC, where no other HPV genotypes were present (sample 16, Table 3), the viral load was relatively low (1/691 viral genomes/No. of human cells). Such minute viral load is consistent with values described previously for other *Betapapillomaviruses* in non-melanoma skin cancer [18], [48], [49], and given the advanced age of the patient (84 years) it is possible that HPV-151 contributed at least partially to the development of the BCC in question. However, further studies are required in order to finally establish the causative role of HPV-151 in the development of BCC of the skin.

Interestingly, one sample of BCC of the skin (sample 8, Tables 2 and 3) contained both novel HPV genotypes: HPV-150 and HPV-151, but not other HPV genotypes. Similarly, one sample of SCC of the skin (sample 6, Tables 2 and 3) contained both HPV-150 and HPV-151 in addition to HPV-37. In both skin cancers, the viral loads per number of human cells were substantially higher for HPV-151 than for HPV-150, but the significance (if any) of these findings remains to be determined.

In addition to full characterization of two novel HPV genotypes, a putative novel HPV genotype, phylogenetically related to HPV-150, was identified in a tissue sample of SCC of the skin obtained from a 64-year old male immuno-compromised patient, together with HPV-9, the type species genotype of *Betapapillomavirus* species 2. The 354-bp sequence of L1 gene of this putative novel HPV genotype (isolate SI-HPV-Beta5; GenBank Acc. No. FR822732) showed 90.68% nt identity with the corresponding part of the L1 sequence of HPV-150 but its other characteristics remain to be determined in future studies.

### Conclusions

The cloning and full characterization of two novel *Betapapillomaviruses* HPV-150 and HPV-151, as well as the partial identification of a putative novel HPV genotype closely related to HPV-150 (isolate SI-HPV-Beta5), improves our knowledge of the diversity of *Betapapillomaviruses* infecting humans. HPV-150 and HPV-151 belong phylogenetically within the *Betapapillomavirus* genus and are most closely related to HPV-96 and HPV-22, respectively. Both novel genotypes are relatively rare, most likely lack mucosal tropism and have clear cutaneous tropism. HPV-150 can establish persistent infection of hair follicles in immuno-competent individuals. HPV-150 and HPV-151 have been found in sporadic cases of common warts, SCC and BCC of the skin preferably in elderly immuno-competent individuals, as both single and multiple infections with viral loads ranging from 1/12 to 1/57,950 viral genomes/No. of human cells.

## Supporting Information

Table S1 Primers used for initial amplification, primer walking and preparation of HPV-150 and HPV-151 reference clones. Nt – Nucleotide position. N – Random nucleotide. Cw – Clock-wise. Ccw – Counter clock-wise.
(DOC)
