# Supplementary material for: Characterization of Novel Cutaneous Human Papillomavirus Genotypes HPV-150 and HPV-151
Source: PLoS One. 2011 Jul 25;6(7):e22529. doi: 10.1371/journal.pone.0022529 (PMC3143161; doi:10.1371/journal.pone.0022529)
Supplement: Table S1 — Primers used for initial amplification, primer walking and preparation of HPV-150 and HPV-151 reference clones. Nt – Nucleotide position. N – Random nucleotide. Cw – Clock-wise. Ccw – Counter clock-wise. (DOC) [file pone.0022529.s001.doc]

Table S1: Primers used for initial amplification, primer walking and preparation of HPV-150 and HPV-151 reference clones.

| **HPV-150** | | **Sequence (5’- 3’)** | **Genomic position (nt)** | **Direction of primer (5’-3’)** |
| --- | --- | --- | --- | --- |
| **Primers used for the initial amplification** | | | | |
| X1-longF | | CTCTCCAGATAATCCAATTAGCGAC | 7097-7121 | cw |
| X1-longR | | GGCTCCAAAGGTACAATACATAGTTG | 7002-7027 | ccw |
| **PCR primers used for cloning** | | | | |
| X1-F5 | | CAGACAGAAATGATTGGGAAAGAAG | 749-773 | cw |
| X1-R5 | | CGTGATGGTTTTTGAAGGAATAAG | 5100-5123 | ccw |
| X1-F18 | | CAACACCTCCCAGGCGAAC | 4985-5003 | cw |
| X1-R18 | | ACAGCAACAGATTCTCCAAACAAC | 990-1013 | ccw |
| **Sequencing primers** | | | | |
| X1fw.2 | CGAATGTTGACGAAGGAG | | 1453-1470 | cw |
| X1rev.2 | TCTGGTCAGCAATTGTTG | | 4376-4393 | ccw |
| X1fw.3 | AATATGTGAGATTCCAAG | | 2245-2262 | cw |
| X1rev.3 | TGCAGGAGCTCCTGATAC | | 3604-3621 | ccw |
| X1fw.4 | TAAAGATGAACCTTGGAC | | 3066-3083 | cw |
| X1rev.4 | TTCTTGGTCACTTAGGTC | | 2775-2792 | ccw |
| X1fw.5 | CCGACCAAGCCACTGCAC | | 3674-3691 | cw |
| X1rev.5 | ATCCATTTGGACATACTC | | 2174-2191 | ccw |
| X1fw.6 | GAACTAAGCGTGATTCTG | | 4286-4303 | cw |
| X1rev.6 | ATATTTCCTACAAAGTTC | | 1584-1601 | ccw |
| X1fw.7 | GCAGATCAAGTGGTAGTG | | 4870-4887 | cw |
| X1rev.7 | GAATTCCCAATCGAGAAG | | 972-989 | ccw |
| X1-F3 | GCTACCAGATGTCCAGACAAAGTAACAC | | 7146-7173 | cw |
| X1-R3 | CATTCCTCTAACAGCCCATACCAAC | | 6137-6161 | ccw |
| X1-F1 | TTGTTGGGGTAATCAAATCTTTT | | 6839-6861 | cw |
| X1-R1 | GGGATCTGATTTTTCCTTTGG | | 7173-7193 | ccw |
| X1-F5 | CAGACAGAAATGATTGGGAAAGAAG | | 749-773 | cw |
| X1-R5 | CGTGATGGTTTTTGAAGGAATAAG | | 5100-5123 | ccw |
| X1-longR | GGCTCCAAAGGTACAATACATAGTTG | | 7002-7027 | ccw |
| **HPV-151** | **Sequence (5’- 3’)** | | **Genomic position (nt)** | **Direction of primer (5’-3’)** |
| **Primers used for the initial amplification** | | | | |
| HPV22-L1-f1mix | | TGGCTTCCANCNTCNGGTAAG | 5686-5706 | cw |
| HPV22-L1-r1mix | | GGTAATAGATGTCAGTNCTNTCCAC | 5761-5785 | ccw |
| X2-1Lfor | | CCGAAACACAAAAAGAAGACCC | 6995-7016 | cw |
| x2-longR2 | | GGTAATAGATGTCAGTTCTCTCCAC | 5761-5785 | ccw |
| **PCR primers used for cloning** | | | | |
| X2-F3 | | GTCAACACCAGTAACCGCACC | 7298-7318 | cw |
| X2-R3 | | TCAACATAATCTTCCAATAACCAATC | 5367-5392 | ccw |
| X2-F17 | | GAGGAGCCACCCGACAGAGAC | 5025-5045 | cw |
| X2-R17 | | GAAGCATACGCACACCCACTG | 403-423 | ccw |
| **Sequencing primers** | | | | |
| X2-L2F1-1 | | TATTTGCCTCCTACGCCACC | 5710-5729 | cw |
| X2-LongR | | GAGTCGCCATACACATCATTAGAC | 6390-6413 | ccw |
| X2-F1 | | GTTACAGTGGCTGATAACACACG | 6688-6710 | cw |
| X2-R2 | | CACTGTGCTACTATCACTGGC | 6736-6756 | ccw |
| X2-R1 | | GGGTCTTCTTTTTGTGTTTCGG | 6995-7016 | cw |
| X2-for2 | | GTTCTTGAAGAGCTTGTC | 676-693 | ccw |
| X2-rev2 | | CACTACGTCTAAATGGAG | 4600-4617 | ccw |
| X2-for3 | | TTAAAGACGCATATGGAG | 1443-1459 | cw |
| X2-rev3 | | CCAGGTTGTGCTGATAAG | 3840-3857 | ccw |
| X2-for4 | | TTCAGCATGTCCCTAATG | 2258-2275 | cw |
| X2-rev4 | | TTACATTTTCAGGATCTC | 3061-3078 | ccw |
| X2-for5 | | GTAGAAACCAGTTTGGAG | 2976-2993 | cw |
| X2-for6 | | CCGCTCCCGATCGCGATC | 3614-3631 | ccw |
| X2-for7 | | TAAAATATGGCAGTGTTG | 4219-4236 | cw |
| X2-for8 | | CAATTGAGAGAATTAGTC | 4834-4851 | cw |
| X2-rev5 | | ATCATCTAAAAGGGCAAG | 2354-2371 | ccw |
| X2-rev6 | | ATAATGCAGCTATCATAC | 1740-1757 | ccw |
| X2-rev7 | | CCTCACTTTGCTGTTGAC | 1128-1145 | ccw |
| X2-rev8 | | AGCAAATCAAGAGACTTC | 535-552 | ccw |
